# Rural Clinical Training for Australian Medical Students: A Scoping Review of Rural Workforce Outcomes

**DOI:** 10.1111/ajr.70243

**Published:** 2026-08-17

**Authors:** Heath Pillen, Debra Jones, Justin Gladman, Lucie Walters, Rowena Ivers, Stuart Lane, Annemarie Hennessy, Xiang‐Yu Hou

**Affiliations:** ^1^ Broken Hill University Department of Rural Health, Faculty of Medicine and Health The University of Sydney Broken Hill Australia; ^2^ Susan Wakil School of Nursing and Midwifery, Faculty of Medicine and Health The University of Sydney Sydney Australia; ^3^ Adelaide University Rural Health Adelaide University Adelaide Australia; ^4^ Graduate School of Medicine, Faculty of Science, Medicine & Health University of Wollongong Wollongong Australia; ^5^ Faculty of Medicine and Health The University of Sydney Sydney Australia

**Keywords:** medical education, rural health academic centres, rural health education and training, rural health workforce development, rural internship

## Abstract

**Introduction:**

Following evidence that participation in rural clinical training during medical school is associated with later rural work, there is a growing literature comparing the effectiveness of different approaches to training.

**Objective:**

To map literature examining the effectiveness of rural clinical training for graduates of Australian medical schools, accounting for differences in training design.

**Design:**

A scoping review was conducted using literature searches performed across five academic databases from January 1, 2000 to January 12, 2026, selecting literature reporting on outcomes related to rural practice intent, internship preferencing or acceptance, and practice location at any point following participation in rural training during medical school.

**Findings:**

A total of 413 unique records were screened, with 43 satisfying the inclusion criteria. Most studies examined outcomes of practice location spanning postgraduate years 1–17 (*n* = 33), with a smaller number examining internship preferencing and/or location (*n* = 10), rural practice intent (*n* = 6) and choice of rural vocational training (*n* = 1). Studies examined the effects of training duration, remoteness of placements, timing and use of longitudinal integrated clerkships (LICs) or other pedagogical approaches.

**Discussion:**

Existing literature has focused on duration‐related effects, consistently identifying that rural training of ≥ 1 year duration is associated with positive rural workforce outcomes in a dose‐dependent manner, and established evidence for the effectiveness of rural LICs, particularly when combined with additional rural training.

**Conclusion:**

There are opportunities to extend from existing literature to establish the effectiveness of core design elements within longer‐duration rural training and LICs within more remote contexts, accounting for interaction with contextual variables.

## Abstract (highlights)

**What Is Already Known on This Subject:**

- Single and multi‐institutional studies have demonstrated that participation in extended rural clinical training (duration ≥ 1 year) and longitudinal integrated clerkships (LICs) is positively associated with rural practice outcomes for graduates of Australian medical schools.
- However, the body of research examining the effectiveness of rural clinical trainings has grown and become increasingly differentiated, creating difficulties in characterising the current evidence base.

**What This Paper Adds:**

- Confirms that the duration of rural training is an important design element contributing to its effectiveness, and that the combination of LICs and additional rural training is consistently associated with improved rural workforce outcomes compared with either approach alone.
- Identifies limitations in knowledge about the effectiveness of extended rural training and LICs when delivered in smaller rural towns, and opportunities to develop understanding of interactions occurring between rural training, contextual factors, and workforce outcomes.

**Practitioner Points:**

- Investment in rural medical training programs is warranted, particularly for Rural Clinical School training ≥ 1 year in duration and for programs that combine LICs with additional rural training experiences.
- Medical students who undertake rural LICs in their penultimate year should be prioritised for rural placements in their final year. This placement sequencing seems to have significant additional benefits for rural workforce outcomes.
- There is a need to consider what other design elements might motivate medical students to work in rural areas across carer stages.

## 1 Introduction

For decades, the geographical maldistribution of Australia's medical workforce has been a persistent and complex policy challenge, with profound consequences for health equity and service access in rural, regional, and remote communities. A reduction in the availability of medical practitioners per head of population is observed with increasing level of remoteness from metropolitan areas. In 2022, there were 427 clinical full‐time equivalents per 100 000 population present in metropolitan areas, compared with only 285 in outer regional and remote areas [1]. This occurs on a background of increasing burden of disease with increasing level of remoteness [2] and an aging rural medical workforce [3]. The imbalance between workforce supply and community need translates into reduced service availability, longer waiting times, travel burdens for patients, and ultimately poorer health outcomes for regional, rural, and remote populations [4].

Since the first Australian National Rural Health Strategy was published in 1994, priority has been placed on recruitment and retention of a rural health workforce, including through reforms to programs of education and training to better align with the needs and circumstances of rural communities [5]. Established from the late 1990s, Australian Rural Clinical Schools (RCSs) and University Departments of Rural Health (UDRHs) have been tasked with enacting these educational reforms in partnership with metropolitan‐based schools and faculties and regional stakeholders, focusing on providing ‘effective’ and ‘quality’ rural training experiences for medical, nursing, and allied health students [6]. The question that confronts RCSs and UDRHs is to what extent they add value to student learning and development, in a way that contributes to rural workforce participation beyond what is expected through metropolitan training alone. Through the lens of educational effectiveness research, there is need to investigate whether these educational exposures ‘directly or indirectly explain measured differences (variations) in the outcomes of students’ [7].

Australian medical schools and medical education researchers have responded to the challenge of building evidence of training or educational effectiveness through establishment of multiple workforce outcome surveys, including the national Medical Schools Outcomes Database (MSOD) survey, rural‐specific Federation of Rural Australian Medical Educators (FRAME) survey, and Medicine in Australia: Balancing Employment and Life (MABEL) survey (2008–2018). Concurrently, pedagogical and organisational developments have occurred within rural medical training, emphasising longer periods of rural training [8], different combinations of block rotation placements and/or longitudinal integrated clerkships (LICs) [9], investment in regional training footprints and end‐to‐end training [10], and developing rural training pipelines that extend across secondary schooling, medical school, and prevocational and vocational training pathways [11]. Together, these developments have contributed to a diverse literature assessing the impact of different approaches to rural medical training on rural workforce outcomes [8].

As rural medical educators move towards a new funding cycle within the Rural Health Multidisciplinary Training (RHMT) Program, there is opportunity to take stock of existing evidence for the educational effectiveness of rural clinical training with respect to the design of training. The present study sought to map available empirical evidence on the effectiveness of rural clinical training offered through Australian medical schools. Effectiveness within this review refers to measured differences in student/graduate outcomes of rural practice intent and rural place of practice that can be explained through differences in student exposure to rural clinical training environments, including considerations of place, duration, clinical education models and pedagogical approaches used, and other identified properties.

## 2 Materials and Methods

### 2.1 Study Design and Framework

This literature review used a scoping review methodology to retrieve and synthesise relevant literature, adhering to reporting guidelines for scoping studies (PRISMA‐ScR) [12]. A scoping review methodology was appropriate for the current study because it allowed for the organisation of diverse literature around concepts related to the design of rural clinical training [13].

### 2.2 Search Strategy

A comprehensive literature search was conducted on 12 January 2026 using academic databases of PubMed, CINAHL, Scopus, Web of Science and ERIC. A search string was developed using keywords and synonymous terms related to ‘clinical training’, ‘rural, regional, and remote’ places of practice in Australian states and territories, ‘medical students’, and workforce and educational outcomes, which were translated into the syntax of each database (see Appendix S1). Reference checking was performed using evaluation of the RHMT Program [14], literature reviews identified through the search, and by scanning the reference lists of included studies.

### 2.3 Inclusion and Exclusion Criteria

Empirical, peer‐reviewed studies published between January 2000 and January 2026 were included in this review if they:

- included students or graduates of Australian medical schools who undertook rural clinical training of more than 8 weeks in a defined non‐metropolitan region, excluding studies of short rural exposures, rural orientation weeks, compulsory short‐term rural rotations, or non‐clinical rural exposure activities;
- reported on outcomes related to rural practice intent, internship preferencing or acceptance, or work location (principal place of practice), measured at any point following completion of rural clinical training, exit from medical school, or any time post‐graduation; and
- provided (quantitative) analytical comparison between training design elements, including but not limited to location (metropolitan versus rural, and differing levels of remoteness), training duration, pedagogical approaches, training site types, or added curricula.

Within this review, clinical training is taken to refer to training provided through hospital and primary care environments where there is opportunity to apply theoretical knowledge (developed through classroom based or pre‐clinical education) to real‐world patient care. Clinical training can be classified as ‘rural’ if it takes place within non‐metropolitan areas according to any classification. In an attempt to differentiate extended rural training from limited short‐term rural rotations, this review only considered the effects of rural placements of ≥ 8 weeks duration [15] delivered within a single non‐metropolitan geographical region, either alone or in combination with clinical training in metropolitan or other rural areas. Appendix S1 reported (rural) practice location was a primary outcome of interest for this review, the review also considered outcomes related to rural practice intent. Despite difficulties in assessing authentic intent, the correlation between rural practice intent at exit from medical school and rural practice location [16] warranted its inclusion as an intermediate outcome of interest. Given that rural workforce outcomes may occur years after an individual graduates from medical school and completes prevocational and vocational training, rural practice intent provides early insight into possible distal workforce effects and supports an analysis of outcomes from newer rural medical training programs. Non‐empirical publications (e.g., commentaries, editorials, letters), studies focused solely on nursing or allied health students, and studies conducted outside Australia were excluded from this review. A detailed list of inclusion and exclusion criteria is provided in Table 1.

**TABLE 1:** Review inclusion and exclusion criteria.

Criteria	Inclusion criteria	Exclusion criteria
Population	Graduates from Australian medical schools Current students attending Australian medical schools Includes both domestic and international student intakes	Graduates from non‐Australian medical schools (overseas trained medical graduates)
Educational intervention	Rural clinical training of any model provided through an Australian medical school Total period of clinical training of > 8 weeks duration within a single defined rural geographical region, which may encompass a mixture of regional, rural, and remote areas.^a^ Examples include the Darling Downs region in Queensland, or Loddon Mallee region within Victoria	Short‐term rural^a^ exposures without a clinical training component, such as ‘rural/country weeks’ Clinical placements of ≤ 8 weeks duration within a single regional, rural, or remote geographical region
Outcomes	Intention to practise in a regional, rural, or remote area^a^ (rural practice intent) at any time point following competition of rural clinical training, reflected for example in data collected from medical school exit surveys and internship preferences Rural^a^ practice location/primary place or practice retention at any time point following graduation from medical school (postgraduate year ≥ 1)	Studies reporting on rural, regional, remote, or country workforce outcomes without reference to accepted^a^ or otherwise well‐defined classifications of rurality or remoteness
Study design	Quantitative study designs, experimental or observational, that include analytical comparison between two or more educational exposures related to educational design (e.g., rural vs. metropolitan training, shorter vs. longer duration, comparison of training approaches)	Descriptive studies Studies focused on the outcomes of a student cohort completed a single educational program, without comparison to metropolitan‐only or alternative rural training Non‐empirical publications, including commentaries, editorials, study protocols, and descriptive articles

^a^ Rurality is defined as any Australian regional, rural, or remote setting, at the time point of classification, according to Modified Monash Model (MMM) categories of MM 2–7, Australian Statistical Geographical Standard/Classification Remoteness Areas (ASGS‐RA) 2–5, or Rural, Remote and Metropolitan Areas (RRMA) 3–7.

### 2.4 Study Selection

After removing duplicate records, the Covidence systematic review tool was used to facilitate a two‐step screening and assessment process to select eligible literature. Title and abstract screening were independently performed by two reviewers (H.P., X.H.), with conflicts managed through reviewer discussion and clarification of eligibility criteria. A sample of 10 full‐text articles was then independently assessed by the same two reviewers, with the remainder assessed by a single reviewer (H.P.). Consistent with guidelines for conducting scoping reviews [13], no quality assessment of studies was performed given the review's aim to map the breadth of available literature.

### 2.5 Data Extraction and Synthesis

Data extraction was performed using a structured template based on the Joanna Briggs Institute Manual for Evidence Synthesis [13]. Studies were grouped according to training design variables including: (i) location of clinical training (metropolitan compared with rural, or differing levels of remoteness), (ii) duration of training, (iii) model of training and (iv) organisation of training elements. These design variables were considered according to their impact across the medical career, from rural practice intent on exit from medical school through to Fellowship.

## 3 Results

### 3.1 Search Results

**FIGURE 1:**
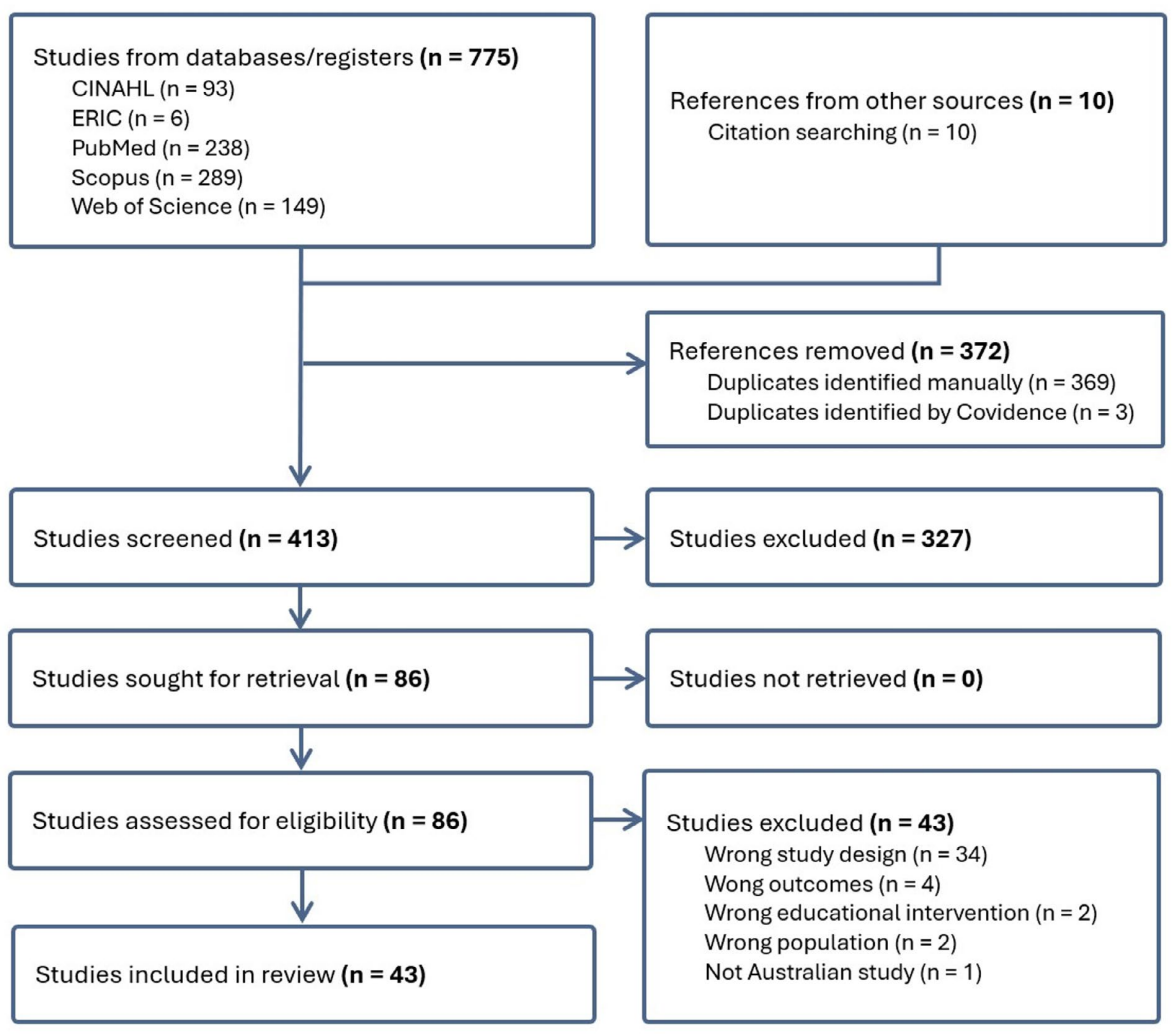
PRISMA diagram of the study selection process, exported and adapted from the Covidence systematic review tool.

After duplicates were removed, 413 records were screened for relevance by title and abstract, and 86 full‐text articles assessed for eligibility. The final synthesis included 43 empirical studies. An overview of the study selection process is described in the PRISMA flowchart in Figure 1. Table 2 provides an overview of key study characteristics for reviewed literature. Of the 43 studies reviewed, most examined outcomes related to practice location spanning postgraduate years (PGY) 1–17 (*n* = 33), with a smaller number examining outcomes related to internship preferencing and/or location (*n* = 10), rural practice intent or preference (*n* = 4 at the point of graduation from medical school and *n* = 2 during later medical practice) and electing rural vocational training (*n* = 1). Most studies examined outcomes for a single medical school or institution (*n* = 28), while others examined outcomes across multiple schools (*n* = 12) or compared outcomes from a single medical school to a national cohort (*n* = 3). The largest body of research related to rural training delivered in Queensland (*n* = 12), followed by nation‐wide cohorts (*n* = 8) and training delivered in Victoria (*n* = 8), Western Australia (*n* = 7), Northern Territory (*n* = 5), New South Wales (*n* = 4), South Australia (*n* = 3) and Tasmania (*n* = 1).

**TABLE 2:** Characteristics of literature reporting on workforce outcomes from rural clinical training for students from Australian medical schools.

Article citation	Educational variables of interest	Outcome of interest	Sample	Location of rural training	Research methodology
Beattie et al. 2024	1‐year rural LIC + 1‐year RCS or 1‐year rural LIC + 1‐year metropolitan training or 2‐year RCS	Practice location, MM2 and MM3–7 (PGY 5–12)	*n* = 932 Graduates of Deakin University medical school between 2011 and 2018	Regional and rural Victoria	AHPRA registry data (2023) linked to training records (single institution)
Campbell et al. 2019	1‐year LIC or other RCS training of similar duration	Practice location (PGY 1–9)	*n* = 2412 Graduates of Monash University medical school between 2008 and 2016	East Gippsland, Victoria	AHPRA registry data (2017) linked to training records (single institution)
Clark et al. 2013	Extended Rural Placement (ERP) ± other RCS training	Rural internship (preference and acceptance)	*n* = 448 Graduates of Sydney Medical School (2005–2010)	Central‐west & far‐west NSW, North Coast NSW	Comparison of MSOD entry/exit survey data (single institution)
Eley, Baker, et al. 2009	1‐year or 2‐years RCS	Practice location	*n* = 124 Graduates of University of Queensland RCS between 2002 and 2006	South‐west and central Queensland	Cross‐sectional survey (single institution)
Eley, Zhang, et al. 2009	1‐year or 2‐years RCS	Internship location	*n* = 812 Graduates from University of Queensland medical school (including UQRCS) and James Cook University	Regional Queensland	Queensland Health Internship register (2007–2008) linked to records of RCS participation for a two Queensland institutions
Forster et al. 2013	1‐year or 2‐years or 3‐years RCS	Current, preferred, and intended place of practice	*n* = 214 Graduates of UNSW RCS between 2003 and 2010	Regional, rural and remote NSW	Cross‐sectional survey (single institution)
Fuller et al. 2021	1‐year RCS + 1‐year rural LIC or 2‐year RCS	Practice location	*n* = 948 Graduates of Deakin University medical school between 2011 and 2018 with Australian practice location	Regional and rural Victoria	AHPRA registry data (2019) linked to training records (single institution)
Fuller et al. 2025	1‐year RCS + 1‐year rural LIC or 2‐year RCS	Practice location—regional footprint or other rural	*n* = 1508 Deakin University medical school between 2011 and 2022 with Australian practice location	Western Victorian training footprint—MM 2–6	AHPRA registry data (2023) linked to training records (single institution)
Isaac et al. 2014	1‐year or ≥ 2‐years RCS	Rural practice intent	*n* = 150 UNSW RCS graduates (2013)	Regional, rural, and remote NSW	Graduate survey—Undergraduate Destination Study (single institution)
Jones et al. 2014	Early (first two years) or late (final two years) rural placements	Rural practice intent	*n* = 3268 Graduates from 21 Australian medical schools with complete MSOD data (graduating from 2005 to 2011)	Australia	National MSOD entry and exist surveys
Kitchener et al. 2015	Rural LIC	Internship location Practice location (post‐internship)	*n* = 683 Graduates from Griffith University medical school participating in RCS Longlook program between 2010 and 2013, and who were employed by Queensland Health through the Queensland Intern Matching Process	Darling Downs & South Burnett, Queensland (RRMA 3–7)	Queensland Health Queensland Intern Matching Process data linked to training records (single institution)
Kitchener 2021	1‐year or 2‐year rural LIC	Internship location Practice location (post‐internship)	*n* = 121 (internship data) and 101 (post‐internship data) Graduates from Griffith University medical school participating in RCS Longlook program between 2010 and 2017	Darling Downs & South Burnett, Queensland (RRMA 3–7)	AHPRA registry data (2018) linked to training records (single institution)
Kondalsamy‐Chennakesavan et al. 2015	1‐year RCS or 2‐year RCS or metropolitan‐only	Practice location	*n* = 754 (from a total population of 2833 medical graduates) Graduates from the University of Queensland between 2002 and 2011, including those from the UQRCS	Regional Queensland	Medical graduates working in rural areas—identified through institutional records and AHPRA registry—who completed a questionnaire between December 2012 and October 2013 (response rate 48%) (single institution)
Kwan et al. 2017	1‐year RCS vs. 2‐year RCS	Rural place of practice (≥ 50% time since graduation)	*n* = 729 Graduates from University of Queensland medical school between 2002 and 2011	Regional Queensland	Cross‐sectional survey (2012) linked to training records (single institution)
May et al. 2018	≥ 1 year RCS	Practice location (PGY 3–5)	*n* = 426 Graduates of University of New England and University of Newcastle medical schools between 2012 and 2014	Hunter New England & Central Coast, NSW	AHPRA registry data (2017) linked to training records (single institution)
McDonnel Smedts and Lowe 2007	17‐week (mean) RCS	Internship location	*n* = 452 Graduates from Flinders University Northern Territory Clinical School between 1999 and 2005	Darwin and rural Northern Territory sites	Institutional training records (single institution)
McDonnel Smedts and Lowe 2008	RCS placement in year 4 (1–19 weeks or ≥ 20 weeks) ± RCS placement in year 3 (40 weeks)	Internship location (Northern Territory vs. other)	*n* = 683 Medical graduates who completed at least one placement within the Northern Territory Clinical School between 1998 and 2007	Northern Territory	Logistic regression analysis of weeks spent training in the Northern Territory against the binary category of return or non‐return for an internship in the Northern Territory
McGirr et al. 2019	≥ 1‐year RCS	Practice location (PGY‐5)	*n* = 1598 Graduates from 12 Australian medical schools in 2011	Australia	AHPRA registry data (2017) linked to training records (12 medical schools)
McGrail et al. 2018	12‐month RCS or 18–24‐month RCS	Practice location (return‐to‐region of training or secondary schooling)	*n* = 2451 Graduates from Monash University medical school	Loddon Mallee and Gippsland, Victoria	AHPRA registry data (2017) linked to training records (single institution)
McGrail and O'Sullivan 2021	< 12 weeks or 3–12 months or ≥ 1‐year rural placement	Practice location (relative to place of secondary schooling and medical training)	*n* = 6627 Graduates from Australian medical schools and University of Queensland medical school	Australia (MABEL)	National MABEL survey (2017) and institutional UQMedCoS survey
McGrail, Gurney, et al. 2023	< 12 weeks or 3–12 months or ≥ 1‐year rural placement	Practice location Proportion of postgraduate time spent in rural practice	MABEL: *n* = 1651 UQMedCoS: *n* = 478 Graduates from Australian medical schools and University of Queensland medical school	Australia (MABEL) and Queensland (UQMedCoS)	National MABEL survey (2017) and institutional UQMedCoS survey
McGrail, Nasir, et al. 2023	Extended Placement Program (12–16 weeks) in rural/remote areas ±1–2‐year RCS	Practice location	*n* = 2806 Graduates from University of Queensland medical school RCS (2012–2021)	Queensland	AHPRA registry data (2017) linked to training records (single institution)
McGrail et al. 2024	Rural placement duration of 0, 1–10, 11–74 or ≥ 75 weeks	Internship (preferencing and acceptance) Continued rural practice at PGY 2–3	*n* = 5956 Graduates from James Cook University, University of Queensland, Griffith University and Bond University medical schools	Queensland hospitals (MM 2–7)	Institutional administration data (2014–2021) from multiple medical schools (JCU, UQ, Griffith, Bond)
O'Sullivan et al. 2018	< 1‐year or 1‐year or 1–2‐year, or > 2 years RCS Rural hospital placement and/or rural general practice (GP)	Practice location (PGY 1–9)	*n* = 2412 Graduates of Monash University medical school at PGY1–9	Regional and rural Victoria (MM 2–7)	AHPRA registry data (2017) linked to training records (single institution)
O'Sullivan et al. 2019	Extended rural cohort (ERC) or non‐ERC RCS	Practice location (PGY 1–9)	*n* = 2412 Graduates of Monash University medical school at PGY1–9	Regional and rural Victoria (MM 2–7)	AHPRA registry data (2017) linked to training records (single institution)
O'Sullivan and McGrail 2020	Rural placement duration < 12 weeks, 3–12 months, > 1‐year	Practice location	*n* = 3151 Participants in the MABEL longitudinal cohort study. Only the post‐2000 cohort was considered in this review	Australia	National MABEL survey (2017)
Playford and Cheong 2012	1‐year RCS or 6‐week rural placement	Practice location (PGY 1–2)	*n* = 490 Graduates of University of Western Australia Medical School from 2003 to 2007	RRMA 3–5 areas within Western Australia	Western Australia internship allocation lists linked to training records (single institution)
Playford et al. 2014	1‐year RCS	Practice location (PGY 3–10)	*n* = 1017 Graduates from the University of Western Australia medical school that completed year 5 of the program between 2002 and 2009	Western Australia (ASGC‐RA 2–5)	AHPRA registry data (2013) for work location (single institution)
Playford et al. 2015	1‐year rural LIC	Practice location	*n* = 324 Doctors practicing rurally within Western Australia (ASGC‐RA 2–5) in 2014 who graduated from University of Western Australia medical school between1980 and 2011	Western Australia (ASGC‐RA 2–5)	AHPRA registry data (2014) linked to training records (single institution)
Playford and Puddey 2017	1‐year RCS Compared non‐RCS applicants (negative control), RCS applicants who did not enter into training (positive control), and RCS participants	Practice location	*n* = 975 Graduates of University of Western Australia medical school	Western Australia (ASGC‐RA 2–5)	AHPRA registry data (2014) linked to training records (single institution)
Playford et al. 2017	1‐year rural LIC	Practice location (PGY 2–7)	*n* = 508 Graduates from University of Western Australia medical school who completed a MSOD entry survey between 2006 and 2010	Western Australia (ASGC‐RA 2–5)	AHPRA registry data (2016) linked to MSOD data (single institution)
Playford et al. 2021	1‐year RCS	Change or maintenance of rural practice intent at entry to and exit from medical school Practice location	*n* = 547 Graduates (2010–2016) of University of Western Australia medical school with completed MSOD entry and exit surveys	Western Australia (ASGC‐RA 2–5)	AHPRA registry data (2014, 2016–2019) linked to MSOD data (single institution)
Playford et al. 2022	RCS participation (length not specified)	Long‐term (≥ 7 years) vs. short‐term rural work amongst female GPs	*n* = 555 Female GPs participating in rural (ASGC‐RA 2–5) work in Western Australia between 2008 and 2018	Western Australia (ASGC‐RA 2–5)	Western Australian Rural Health West longitudinal workforce dataset with annual linkage (2008–2018)
Seal et al. 2022	RCS ≥ 1‐year	Practice location (PGY 5–8)	*n* = 1321 Domestic students who graduated from (10) Australian medical schools in 2011	Australia (FRAME sites)	AHPRA registry data linked to data from national FRAME survey (multi‐institution)
Seal et al. 2024	RCS ≥ 1‐year	Practice location (PGY‐10), grouped by specialty (non‐fellowed, GP or non‐GP)	*n* = 1220 Domestic students that graduated from one of 9 Australian medical school in 2011	Australia (FRAME sites)	AHPRA registry data linked to data from national FRAME survey (multi‐institution)
Sen Gupta et al. 2013	End‐to‐end rural training with ≥ 20 weeks training in small rural or remote towns	Rural practice intent Internship location	*N* = 292 JCU students, *n* = 720 students from Queensland medical schools, and *n* = 1457 participants from other Australian medical schools Graduates (2005–2010) of James Cook University medical school and eight other medical schools with MSOD data	Queensland (ASGC‐RA 2–5)	Medical school exit survey—administered by JCU (2005–2010) and through the MSOD (2009–2010 for eight metropolitan‐based schools)
Shires et al. 2015	RCS ≥ 1‐year	Practice location	*n* = 974 Graduates from University of Tasmania medical school between 2002 and 2013	Tasmania	AHPRA registry data (2014) linked to institutional records (single institution)
Sureshkumar et al. 2017	RCS exposure (any duration)	Electing a rural training pathway through the Australian General Practice Training (AGPT) pathway	*n* = 1253 Applicants to the AGPT pathway in 2015 for whom complete data was available and who were eligible for either metropolitan or rural training pathways	Australia	AGPT applicant data (2015)
Walker et al. 2021	1‐year Parallel Rural Community Curriculum (PRCC) program (LIC)	Practice location	*n* = 1552 Graduates from Flinders University Medical School who were eligible for the PRCC program (graduating years 1999–2012)	Rural towns in South Australia and Victoria	AHPRA registry data (2017) linked to institutional records (single institution)
Williams et al. 2023	Rural LIC	Practice location	*n* = 2078 Graduates of the University of Adelaide medical school between 2004 and 2019	South Australia	AHPRA registry data (2021) linked to institutional records (single institution)
Woolley et al. 2016	2‐year RCS (site comparison)	Internship location (relative to location of training) Practice location (PGY 4–9) (relative to location of training)	*n* = 620 for James Cook University medical school graduates with known internship location *n* = 406 for James Cook University medical school graduates at PGY 4–9 (graduating between 2005 and 2013)	Northern Queensland (Townsville, Cairns, Mackay) and Northern Territory (Darwin)	Longitudinal workforce tracking between 2006 and 2014 (single institution)
Woolley et al. 2017	2‐year RCS (site comparison)	Remote practice (ASGC‐RA4–5) for ≥ 1 year at any time between PGY 4–10	*n* = 529 Graduates from James Cook University medical school at PGY 4–10	Northern Queensland (Townsville, Cairns, Mackay) and Northern Territory (Darwin)	Cross‐sectional analysis (2015) of longitudinal workforce tracking data (single institution)
Worley et al. 2008	1‐year rural training	Preferred practice location—rural, urban, or both (PGY 6–8)	*n* = 74 Graduates from the Flinders University Parallel Rural Community Curriculum (PRCC), Northern Territory Clinical School (NTCS) and Flinders Medical Centre medical training streams, undertaking their clinical training (year 3) from 1998 to 2000	Northern Territory (Darwin) or rural South Australia (Riverland)	Postal survey (2005) of Flinders University medical school graduates (single institution)

Abbreviations: AHPRA, Australian Health Practitioner Regulation Agency; ASGS‐RA/ASGC‐RA, Australian Statistical Geographical Classification/Standard—Remoteness Area; FRAME, Federation of Rural Australian Medical Educators; LIC, longitudinal integrated clerkship; MABEL, Medicine in Australia: Balancing Employment and Life; MM, Modified Monash Model category; MSOD, Medical Schools Outcomes Database; PGY, postgraduate year; RCS, Rural Clinical School; RRMA, Rural, Remote and Metropolitan Area classification; UQMedCoS, University of Queensland's Medical Graduates Cohort Study.

### 3.2 Rural Practice Intent After Rural Clinical Training or at Graduation

Of the four studies in this scoping review that examined outcomes of rural practice intent at the point of graduation or completion of rural clinical training, three used the measure of preferred practice location contained within the exit questionnaire of MSOD surveys. One study used MSOD survey data from 21 Australian medical schools (*n* = 3268), comparing the effect of rural and remote placements delivered within the final 2 years of medical school on intention to practice in a regional city/large town or smaller [17]. This study established that rural (RRMA 3–5) and remote area (RRMA 6–7) placements in the final 2 years of medical school were associated with greater rural practice intent (working in a regional city/large town or smaller) after controlling for demographic and school‐level characteristics.

Examining data from a single‐institution, Playford et al. [16] examined both MSOD entry and exit scores to determine whether RCS training of 1‐year duration was effective in changing or maintaining urban/rural practice intent, analysed separately for rural and urban‐background students. RCS training was found to produce a fourfold increase in the likelihood of urban‐background students changing from urban to rural practice intent at exit from medical school. Although non‐statistically significant associations were observed between RCS‐participation and change or maintenance in rural practice intent for rural‐background students, it is recognised that the small size of some analytical categories may have contributed to these non‐significant findings. Sen Gupta et al. [18] identified increased odds of rural practice intent on exit from the regionally‐based James Cook University medical program (featuring ≥ 20 weeks of clinical training in smaller rural or remote towns) when compared to eight other medical schools with available MSOD data (odds ratio [OR] = 4.6, 95% CI: 3.5–6.0). Using an in‐house exit survey assessing post‐vocational rural practice intent amongst students completing 1–4 years of training at a single RCS (*n* = 165), another study found that > 2 years of RCS training was associated with increased odds of intending to work in a regional, rural, or remote area compared to those completing a single year of RCS training [19].

### 3.3 Internship Preference and Acceptance

Ten articles examined the association between rural clinical training, preference for rural internship, and/or internship location. Clark et al. [20] identified that 1‐year RCS training from a single‐institution was associated with greater likelihood of both preferencing and accepting a rural internship, whereas another study found that Queensland interns in RRMA3 locations in 2007 and 2008 were more likely to have attended Queensland‐based RCSs compared to urban‐based schools (unadjusted OR = 8.8, 95% CI: 4.6–16.7 and OR = 6.5, 95% CI: 3.5–12.2 for 2007 and 2008, respectively) [21]. Bivariate analyses of workforce outcomes have also found that ≥ 1 year RCS training was significantly associated with increased likelihood of rural practice (MM2‐7) for > 40% of a graduates prevocational period [22].

Considering return‐to‐region outcomes, participation in the Northern Territory Rural Clinical School for 1 year was associated with increased internship acceptance for 70% for quota (Northern Territory and Aboriginal) students and 48% for other RCS students, compared to 4% for metropolitan‐based students [23]. Positive return‐to‐region outcomes have also been observed for the rural end‐to‐end James Cook University medical program, in which training within a given RCS campus was significantly associated with future internship in the same location (and also working in that location in PGY 4–9) [24].

There is also evidence for the benefit of longer rural training *duration* on rural internship preference. A study of graduates from three Queensland medical schools (2014–2021) found that greater length of time spent in undergraduate rural placements (11–74 weeks, and 75+ weeks, but not 1–10 weeks duration) was associated with greater odds of having a first preference for rural internship (MM 2–7), and that this was true for both pre‐ and post‐regional training hub student cohorts [25]. Another study compared shorter (6‐week Rural Undergraduate Support and Coordination‐funded) rural placements to 1‐year RCS training, with the latter associated with greater odds of rural work location (RRMA 3–5) at PGY‐1, with the effect on rural work increasing at PGY‐2 (OR = 3.0, 95% CI: 1.6–5.6) [26]. In their analysis of the relationship between placement time spent in the Northern Territory [Rural] Clinical School and Northern Territory internship location, McDonnel Smedts and Lowe [27] identified a positive association between placement length and odds of rural internship (OR = 1.1, 95% CI: 1.07–1.09), with full penultimate year rural training combined with long‐term final year placement within the RCS producing the greatest odds of returning to the Northern Territory as an intern.

The *pedagogical approach* used may also matter. Although unable to separate the effects of rural exposure and effects due to the LIC itself, one study compared the effect of metropolitan block rotations to that of rural LICs, identifying positive associations between rural LIC participation and undertaking rural (RRMA 3–5) internship (OR = 11.9, 95% CI: 6.1–23.3) [28]. Other studies have found that participation in longer *duration* (2 year) LICs is associated with greater odds of internship in rural locations (ASGS‐RA 2–5) (OR = 6.7, 95% CI: 1.8–22.6) [29] and is associated with increased odds of return‐to‐region for internship (OR = 5.7, 95% CI: 2.2–14.8) [30].

### 3.4 Rural Vocational Training Preference or Acceptance

Only one study examined in this review examined the effect of RCS training on choice of vocational training location [31]. This study examined the records of applicants to the Australian General Practice Training (AGPT) program, identifying an association between RCS participation and preference for a rural training pathway (OR = 2.0, 95% CI: 1.5–2.8).

### 3.5 Preference for Rural Practice Amongst Practicing Doctors

Two studies examined preference for rural practice amongst practicing doctors, indicative of an enduring desire for future rural practice. One study examined preferred work location at PGY 4–6 amongst graduates from two RCSs compared to those receiving metropolitan training only, finding a strong preference for rural work amongst those that had completed rural training (OR = 19.1, 95% CI: 3.4–106.3 for the Flinders University Parallel Rural Community Curriculum and OR = 4.3, 95% CI: 1.2–14.8 for the Northern Territory Clinical School) [32]. Indicative of training *duration* effects, 3 years of training within a single RCS was associated with increased odds of wanting to work in a rural location after completion of training, compared with a single year of rural training (OR = 5.1, 95% CI: 1.8–14.2) amongst practicing doctors at PGY 1–8 [33].

### 3.6 Work Location From PGY 1–17

The majority of studies in this review examined work location as the outcome of interest, spanning the period of PGY 1–17. A large body of literature sought to establish whether RCS training, of ≥ 1 year duration, was effective at increasing the likelihood of working in a rural area relative to (standard) metropolitan‐based training. Single‐institution studies that examined rural employment outcomes from RCS training of ≥ 1 year while accounting for student rural origin have identified positive associations between rural training and rural work location at PGY 2–5 (OR = 1.9, 95% CI: 1.1–3.5) [34], PGY 3–5 (OR = 6.1, 95% CI: 2.7–7.5) [35], PGY 3–10 (OR = 7.5, 95% CI: 3.5–15.9 and OR = 5.1, 95% CI: 2.9–9.1 for rural and metropolitan‐background students, respectively) [36], PGY 1–12 (OR = 4.9, 95% CI: 3.2–7.6 for ASGS‐RA 3–5 and OR = 9.0, 95% CI: 4.7–17.2 for MM 2–7) [37] and PGY 1–17 (OR = 3.1, 95% CI: 2.1–4.5) [38]. Likewise, Kwan et al. [39] identified that long‐term rural practice (rural work location for ≥ 50% of time since graduation) was associated with 1 or 2 years of RCS training, with the greatest effect size observed for longer‐duration training for those with a rural background (OR = 10.4, 95% CI: 4.9–21.9 for 2 years of RCS training and rural background). Comparing rural employment outcomes (ASGS‐RA 3–5) with RCS participation for ≥ 1 year relative to pre‐RCS student cohorts, Playford et al. also [40] identified increased odds of rural employment (OR = 4.1, 95% CI: 2.0–8.3) after adjusting for sex and rural origin. Playford et al. also examined the influence of gender, reporting a large but statistically insignificant association between RCS participation and rural employment at PGY ≥ 8 years for female medical graduates in Western Australia [41].

In their analysis of rural (ASGS‐RA 2–5) employment amongst RCS graduates at PGY‐3 or beyond, Playford and Puddey [42] found that those who preferenced and participated in RCS training had the greatest odds of rural practice, particularly in outer regional and remote (ASGS‐RA 4–5) areas (OR = 5.4, 95% CI: 2.2–13.5). Campbell et al. [43] also found that RCS training > 1 year increased the likelihood of having a current work location (PGY 1–9) in large regional centres (RRR = 4.5, 95% CI: 2.8–7.2, MM‐2) and smaller rural towns (Relative Risk Ratio (RRR) = 3.0, 95% CI: 1.9–4.8, MM 3–7), and Woolley et al. identified that participation in a given RCS (in Darwin, but not other northern Australian RCSs) was associated with increased odds of practicing for ≥ 1 year in a remote town between PGY 4–10 [44].

Analysing data from 14 Australian medical schools, McGirr et al. [45] found that practitioners that had trained for ≥ 1 year in a RCS had greater odds of being in rural practice at any point post‐graduation (OR = 1.5, 95% CI: 1.2–2.1, for ASGS‐RA 2–5 and OR = 2.6, 95% CI: 1.8–3.8 for MM 3–7). Using data from 10 Australian medical schools, Seal et al. [46] reported similar findings, with RCS participation of ≥ 1 year associated with greater likelihood of practice in regional (MM‐2) or rural‐remote (MM 3–7) communities at PGY‐8 for both metropolitan and rural origin practitioners, with the greatest effect size observed for students of rural origin working in rural communities (RRR = 4.8, 95% CI: 3.1–7.5). Further analysis of medical graduates from across nine medical schools found that the odds of working in rural general practice (GP) (MM 2–7) in PGY‐5 and PGY‐10 was significantly greater for RCS graduates, and that RCS participation had a positive but non‐significant association with work as a non‐GP specialist in a rural location [47].

Examining the likelihood of medical graduates returning to work in their region of training, McGrail and O'Sullivan [11] drew on a national cross‐sectional survey of 6627 Australian doctors (MABEL), finding that those who completed ≥ 1 year rural training were more than 5 times more likely than those who had < 12 weeks of rural training to be working in their same region of training (RRR = 5.2, 95% CI: 4.0–6.9), increasing for those who also spent their childhood (≥ 6 years) in the same rural region (RRR = 17.4, 95% CI: 11.6–26.1, compared to RRR = 2.7, 95% CI: 1.8–4.1 for working in any rural region). Even for metropolitan origin students, RCS participation of ≥ 1 year was significantly associated with return to region (RRR = 7.0, 95% CI: 4.9–9.8) or working in a different rural region. A study examining work location for graduates of the end‐to‐end James Cook University medical program demonstrated that graduates were also more likely to work in their region of training at PGY 4–9 [24].

Extending from literature examining the effectiveness of clinical training delivered through rural campuses, a sizeable body of literature has examined the relationship between the *duration* of rural clinical training and work location. Two studies compared shorter clinical training to either no or year‐long RCS training [48, 49]. In one study [48], sub‐group analysis of practice location following participation in an optional 6–8 week rural clinical training extension (in addition to earlier compulsory rural clinical training) identified a positive but non‐significant association between the additional rural placement and practising in a rural location (MM 2–7) at PGY 1–10 (OR = 2.0, 95% CI: 0.9–4.1) when compared to no extended placement participation. In analysis of MABEL data (2017) for doctors who graduated from medical school from 2000 to 2017 [49], an association was observed between rural training of 3–12 months and current rural practice location (MM 2–7) (OR = 1.4, 95% CI: 1.0–2.0) after adjusting for sex, rural origin, and GP speciality. However, the study reported a larger effect size for rural training > 1 year in duration (OR = 2.3, 95% CI: 1.6–3.3), and for an increasing number of rural placements.

Other studies have examined the effect of RCS training extending beyond 1 year duration. O'Sullivan et al. [50] identified a dose–response relationship between years of RCS training and rural work location (MM 2–7) at PGY 1–9, with the greatest odds of rural practice (MM 2–7, MM‐3 and MM 2–7) associated with > 2 to 3 years RCS training. McGrail et al. [48] also identified that RCS participation for 1 or 2 years was associated with greater odds of practicing in a rural area (MM 2–7) at any time after graduation, with the greatest odds observed with 2 years RCS training (OR = 2.8, 95% CI: 2.2–3.6). Similarly, Kwan et al. [39] identified that 1 and 2 years of RCS training were associated with increased odds of rural practice (ASGS‐RA 2–5) for ≥ 50% of time since graduation for general practitioners and (other) medical specialists. A similar dose–response relationship has been observed for rural practice (ASGC‐RA 2–5) amongst medical graduates (PGY 1–10) completing 1 and 2 years RCS training [51], although this study only identified a significant association for students with rural background (OR = 4.4, 95% CI: 2.4–8.3 and OR = 7.1, 95% CI: 3.6–14.1 for 1 and 2‐years RCS training, respectively). Although Forster et al. [33] identified a positive association between current work location and longer RCS training (particularly 3 years compared to 1 year of rural training) for a single institution, this association failed to reach the threshold for statistical significance (OR = 2.5, 95% CI: 0.9–7.0). Considering return‐to‐region outcomes in PGY 1–9, McGrail et al. [30] identified that RCS training for 18–24 months (compared to 1 year) was associated with greater likelihood of working in the same rural region (RRR = 3.4, 95% CI: 1.9–6.0), but not other rural regions.

A body of literature has also examined the relative effectiveness of different *clinical education models* or *pedagogical approaches*. Studies comparing metropolitan block rotations with rural LICs have identified associations between rural LIC participation and rural work location—including in areas outside of regional centres (OR = 3.1, 95% CI: 2.1–4.4 for MM 3–5 areas, OR = 3.4, 95% CI: 2.1–5.6 for MM 6–7 areas) [52] and for MM 3–7 areas [38]. Other sub‐group analyses of the effect of rural LIC participation in the penultimate year of medical school have identified positive but non‐significant associations between LIC participation and rural practice location at PGY 1–9 in regional (MM‐2) or rural and remote (MM 3–7) areas, with the non‐significant association a possible consequence of student self‐selection effects and the relatively small number of cases used for subgroup analysis [43]. A sub‐set of this literature considers the organisation of rural LICs within a wider program of training. Using metropolitan medical training as a reference, any extended rural clinical training (with or without LIC participation) produced greater odds of rural employment (ASGS‐RA 2–5) from PGY 1–8, with a penultimate rural LIC year and final RCS year producing the greatest effect (OR = 7.1, 95% CI: 3.5–14.2) [53]. Beattie et al. [54] also identified that the greatest odds of working in a rural area (MM2 and MM3–7) came from a combined penultimate‐year LIC and final‐year of RCS training (OR = 4.2, 95% CI: 1.7–10.1 and OR = 5.4, 95% CI: 2.2–13.4, respectively), but not for alternatives of 2‐year RCS training or a 1‐year LIC combined with a final year of metropolitan clinical training. Other research has identified that the odds of a medical graduate working in their same region of training (for the training footprint of a single medical school) were greatest for those participating in a 1‐year LIC with a further 1‐year RCS training (OR = 6.8, 95% CI: 2.9–15.7), followed by those receiving 2‐year RCS training (OR = 4.1, 95% CI: 2.6–6.4) [55].

The effectiveness of other approaches to curricular design has also been examined. O'Sullivan et al. [50] identified that the combination of clinical training in regional hospitals and in rural GP, relative to either approach alone, increases the likelihood of medical graduates working in a rural location (MM 2–7) at PGY 1–9 after accounting for duration of rural training (RRR = 3.7, 95% CI: 2.5–5.3). When compared to metropolitan‐only training, participation in the geographically contained Extended Rural Cohort (ERC) program was found to increase the likelihood of medical graduates working in a large regional area (MM‐2), but not for rural towns (MM 3–7) [56]. In the same study, RCS participation of either 1 to < 2 years or 2–3 years duration did result in greater likelihood of working in both regional centres and rural towns, with the authors speculating that the RCS pathway enabled greater choice of location amongst medical students, potentially impacting positively on their rural training experiences [56]. McGrail et al. [48] also found that the combination of a 12‐week Extended Placement Programme (EPP) with additional (1–2) years of regional clinical training offered the greatest odds of rural employment at PGY 1–7 when compared to students with no EPP or regional training exposure (OR = 8.6, 95% CI: 4.5–16.3, for those participating in the EPP and 2 years of regional clinical training).

There is limited evidence for the effect of the *remoteness* of clinical training on future work location. This evidence comes from a single study using national cross‐sectional MABEL survey data for doctors graduating from medical school between 2000 and 2017 (*n* = 6510), finding a significant association between completing placements in more remote areas (MM 4–7) and current principal place of practice in any rural area (MM 2–7) (OR = 1.8, 95% CI: 1.6–2.1) [49].

## 4 Discussion

Reviewed studies provide consistent evidence that extended rural medical training contributes significantly to rural workforce outcomes. Although longer training duration does appear to produce greater returns in terms of workforce outcomes in a dose‐dependent manner, the combination of LICs with additional rural training time also appears to have a strong effect that warrants further research across different rural settings. Importantly, these studies show that both rural‐origin and metropolitan students can be positively influenced towards rural practice when provided with these rural training experiences, albeit favouring rural‐origin students and those with rural practice intent at entry to medical school. The evidence of effectiveness for extended (1, 2 and 3‐year) RCS‐based training is particularly robust, with a large body of evidence available from single and multi‐institution studies, drawing associations between RCS participation and a range of educational outcomes, including rural practice intent, internship preference and acceptance, and place of practice spanning PGY 1–17. Although limited evidence was identified in relation to rural end‐to‐end medical education and training, this was a consequence of the eligibility criteria used and its requirement for comparative studies, rather than a lack of evidence for end‐to‐end education and training per se.

The findings of this Australian‐based scoping review are consistent with international literature, which has identified that medical students who attend a rural campus and spend more time in rural areas are more likely to practice in these locations following graduation [57], as are students who complete rural LICs over rotation‐based placements [58]. Participation in extended rural clinical training and LICs is thought to influence career decision‐making through a range of mechanisms, including the development of rural practice self‐efficacy via more active involvement in patient care, identification of compatible role models and/or mentors, greater satisfaction gained through sustained patient and community‐doctor relationships, and the establishment of a grounded understanding of rural practice [59]. However, generalisations about the effectiveness of impact of rural clinical training on workforce outcomes are limited by the recognition that rural career choice is situated, influenced by family and lifestyle considerations and by perceptions of the availability of professional support, career opportunities, and healthcare infrastructure [60]. Therefore, care is likely required when translating models of training from one rural context to another.

Despite these limitations, findings from the current literature review suggest that government investment in longer duration rural training is warranted, and confirm the importance of implementing comprehensive and coherent rural workforce policy at national and state levels [61] together with careful selection of students who are more likely to work in rural areas post‐graduation [16]. However, there are also opportunities to further examine how different training design features interact with the training pipeline (from secondary schooling through to vocational training), health service, and community factors to produce workforce outcomes. Consideration of such interactions will become more important as the training of medical students extends beyond large regional centres and teaching hospitals and into smaller rural and remote towns. This movement will require adaptation of training and supervision models to provide students with sufficient breadth of clinical experience and positive rural experiences, providing opportunities to assess the relative effectiveness of training design elements related to interprofessional learning and teamwork, use of telehealth and other digital health technologies, and simulation‐based training [10].

Although providing an overview of the impact of rural clinical training on workforce‐related outcomes, this scoping review is limited in several ways. Firstly, it is possible that rural training exposures may have been misclassified given the limited description of rural curricula within some published literature, which would have the effect of overlooking or misrepresenting important training design variables and reducing the review's ability to identify more nuanced branches of research. The review's focus on examining educational effectiveness through comparative studies also excluded a body of literature related to end‐to‐end medical education and training. Much of the end‐to‐end medical education and training literature is grounded in theory and research related to the effects of place‐based rural pipelines [62], with a different and more expansive analytical focus compared to our narrower study of rural clinical training exposures during medical school. While our review focused on analytical comparative studies in order to explore the relationship between training design and educational effectiveness, it did exclude other study designs, such as realist evaluations or systems modelling approaches, that could account for interactive effects occurring between training design and student characteristics, school organisation and curriculum, and policy inputs. A sound understanding of how these factors interact would provide a more nuanced understanding of how rural practice intent and career choices might develop or be maintained over time, including circumstances where intent might translate to career choice. Although rural practice intent is a plausible mediator of the relationship between rural clinical training and subsequent rural work location [16], the complex pathway linking intent to career choice [63] means that findings related to rural practice intent should be interpreted with caution. While regression modelling has been used extensively to generate evidence of associations between training exposure and workforce outcomes, drawing from data available in national or institutional surveys and registries, such analyses are limited in their ability to draw causal inferences and make sense of complex educational processes and their effects [7]. The field of education studies offers alternative methodologies for evaluating education or training effectiveness, including multilevel and systems approaches, that may guide future assessment of the effects of rural clinical training.

## 5 Conclusion

There is a large and consistent body of literature identifying associations between rural medical training offered through RCSs and positive rural medical workforce outcomes, particularly for training greater than 1 year duration and where extended rural training is combined with LICs. Further investigation is required to develop understanding of the relative effectiveness of pedagogical approaches nested within extended rural clinical training and LICs when delivered outside of regional centres, and how the effectiveness of these approaches is influenced by community, school/organisation, and post‐graduate training environments.

## Author Contributions

**Lucie Walters:** writing – review and editing. **Justin Gladman:** writing – review and editing. **Debra Jones:** conceptualization, writing – review and editing. **Rowena Ivers:** writing – review and editing. **Annemarie Hennessy:** writing – review and editing. **Stuart Lane:** writing – review and editing. **Xiang‐Yu Hou:** conceptualization, investigation, writing – original draft, writing – review and editing, visualization, methodology, formal analysis, project administration, data curation, supervision. **Heath Pillen:** writing – original draft, visualization, writing – review and editing, formal analysis, project administration.

## Funding

Although this research did not receive specific funding, the positions of authors affiliated with the Broken Hill University Department of Rural Health, The University of Sydney (Heath Pillen, Debra Jones and Xiang‐Yu Hou) are funded by the Australian Government Department of Health through the Rural Health Multidisciplinary Training (RHMT) Program.

## Supporting information


**Appendix S1:** List of database searches (search date: 12 January 2026).

## Data Availability

Data sharing not applicable to this article as no datasets were generated or analysed during the current study.
