# Supplementary material for: Rural Clinical Training for Australian Medical Students: A Scoping Review of Rural Workforce Outcomes
Source: Aust J Rural Health. 2026 Aug 17;34(4):e70243. doi: 10.1111/ajr.70243 (PMC13482001; doi:10.1111/ajr.70243)
Supplement: Supplementary file 1 [file AJR-34-0-s001.docx]

**Supplement 1. List of database searches (search date: 12 January 2026)**

**SCOPUS**

TITLE-ABS-KEY ( ( "student placement" OR "clinical placement*" OR "clinical education" OR placement* OR "practice placement*" OR "clinical experience*" OR "clinical training" OR "rural rotation*" OR "rural training" OR training OR clerkship OR "rural immersion" ) AND ( rural* OR remote OR regional ) AND ( Australia* OR Queensland OR Tasmania OR Victoria OR "New South Wales" OR NSW OR "Northern Territory" OR ACT) AND ( medic* W/5 ( student* OR undergraduate* OR postgraduate* ) ) AND ( ( learn* W/5 (outcome* OR objective*) ) OR ( (regional OR rural OR remote) W/7 intent* ) OR internship OR employ* OR ( work* W/5 (regional OR rural OR remote)) OR recruit* OR retention ) )

**CINAHL**

("Student Placement" OR "clinical placement*" OR "clinical education" OR placement* OR "practice placement*" OR "clinical experience*" OR "clinical training" OR training OR "rural rotation*" OR "rural training" OR clerkship OR "rural immersion" OR MH "Student Placement") AND (rural* OR remote OR regional OR MH "Rural Health Services" OR MH "Rural Health Centers" OR MH "Rural Health Personnel" OR MH "Rural Health" OR MH "Rural Population" OR MH "Rural Areas") AND MH "Australia" AND ((medic* N5 ( student* OR undergraduate* OR postgraduate* ) ) OR MH "Students, Medical+") AND ( ( learn* N5 (outcome* OR objective*) ) OR ( (regional OR rural OR remote) N7 intent* ) OR internship OR employ* OR ( work* N5 (regional OR rural OR remote)) OR recruit* OR retention ) )

**PubMed**

((((("Student Placement"[Title/Abstract] OR "clinical placement*"[Title/Abstract] OR "clinical education"[Title/Abstract] OR placement*[Title/Abstract] OR "practice placement*"[Title/Abstract] OR "clinical experience*"[Title/Abstract] OR "clinical training"[Title/Abstract] OR training[Title/Abstract] OR "rural rotation*"[Title/Abstract] OR "rural training"[Title/Abstract] OR clerkship[Title/Abstract] OR "rural immersion"[Title/Abstract]) AND (rural*[Title/Abstract] OR remote[Title/Abstract] OR regional[Title/Abstract])) AND (Australia*[Title/Abstract] OR Queensland[Title/Abstract] OR Tasmania[Title/Abstract] OR Victoria[Title/Abstract] OR "New South Wales"[Title/Abstract] OR NSW[Title/Abstract] OR "Northern Territory"[Title/Abstract])) AND (medic*[Title/Abstract] AND (student*[Title/Abstract] OR undergraduate*[Title/Abstract] OR postgraduate*[Title/Abstract]))) AND (recruitment OR retention OR "rural health workforce" OR employ* OR intern* OR "postgraduate year" OR "rural intent" [Title/Abstract:~7]))) AND ((learn*[Title/Abstract] AND (outcome*[Title/Abstract] OR objective*[Title/Abstract]) ) OR ( (regional[Title/Abstract] OR rural[Title/Abstract] OR remote[Title/Abstract]) AND intent* [Title/Abstract]) OR internship[Title/Abstract] OR employ*[Title/Abstract] OR ( work*[Title/Abstract] AND (regional[Title/Abstract] OR rural[Title/Abstract] OR remote[Title/Abstract])) OR recruit*[Title/Abstract] OR retention [Title/Abstract]))

**Web of Science**

("Student Placement" OR "clinical placement*" OR "clinical education" OR placement* OR "practice placement*" OR "clinical experience*" OR "clinical training" OR training OR "rural rotation*" OR "rural training" OR clerkship OR "rural immersion" ) AND ( rural* OR remote OR regional ) AND ( Australia* OR Queensland OR Tasmania OR Victoria OR "New South Wales" OR NSW OR "Northern Territory" OR ACT) AND ((medic* NEAR/5 ( student* OR undergraduate* OR postgraduate* ) ) AND ( ( learn* NEAR/5 (outcome* OR objective*) ) OR ( (regional OR rural OR remote) NEAR/7 intent* ) OR internship OR employ* OR ( work* NEAR/5 (regional OR rural OR remote)) OR recruit* OR retention ) )

TS=("medical student*" AND "rural placement*" AND ("workforce retention" OR "rural recruitment"))

**ERIC**

("Student Placement" OR "clinical placement*" OR "clinical education" OR placement* OR "practice placement*" OR "clinical experience*" OR "clinical training" OR training OR "rural rotation*" OR "rural training" OR clerkship OR "rural immersion" ) AND ( rural* OR remote OR regional ) AND ( Australia* OR Queensland OR Tasmania OR Victoria OR "New South Wales" OR NSW OR "Northern Territory" OR ACT) AND ((medic* ADJ5 ( student* OR undergraduate* OR postgraduate* ) ) AND ( ( learn* ADJ5 (outcome* OR objective*) ) OR ( (regional OR rural OR remote) ADJ7 intent* ) OR internship OR employ* OR ( work* ADJ5 (regional OR rural OR remote)) OR recruit* OR retention ) )
